# A novel neutrophil extracellular traps-related lncRNA signature predicts prognosis in patients with early-stage lung adenocarcinoma

**DOI:** 10.1080/07853890.2023.2279754

**Published:** 2023-11-19

**Authors:** Huan Wang, Yueli Shi, Xia Xu, Shumin Xu, Yuting Shi, Weiyu Chen, Kai Wang

**Affiliations:** a Department of Respiratory and Critical Care Medicine, The Fourth Affiliated Hospital, Zhejiang University School of Medicine, Yiwu, China; b Department of Pathology, The Second Affiliated Hospital of Zhejiang University School of Medicine, Hangzhou, China

**Keywords:** Lung cancer, long non-coding RNAs, neutrophil extracellular traps, prognosis signature, FAM66C

## Abstract

**Background:**

Neutrophil extracellular traps (NETs) could entrap tumour cells and promote their dissemination and metastasis. Further analysis of NETs-related molecules is expected to provide a new strategy for prognosis prediction and treatment of lung adenocarcinoma (LUAD) patients.

**Methods:**

The model construction was established through co-expression analysis, Lasso Cox regression, univariate and multivariate COX regression, Gene ontology and Kyoto Encyclopedia of Genes and Genomes pathway. The potential drugs and analysed drug sensitivity were screened by pRRophetic packages.

**Results:**

In this study, we constructed a 15 NETs-related long non-coding RNAs (lncRNAs) prognostic prediction model (AC091057.1, SPART-AS1, AC023796.2, AL031600.2, AC084781.1, AC032011.1, FAM66C, C026355.2, AL096870.2, AC092718.5, PELATON, AC008635.1, AL162632.3, AC087501.4 and AC123768.3) for patients with early-stage LUAD based on public databases and datasets. The signature is associated with immune cell functions, tumour mutation burden and treatment sensitivity in LUAD patients. Additionally, we found that FAM66C is highly expressed in lung cancer patients for the first time, which is associated with poor prognosis. FAM66C knockdown significantly inhibited the proliferation and migration ability of the tumour cells.

**Conclusions:**

In conclusion, this model is a new and effective prognostic and efficacy predictive biomarker, FAM66C plays an oncogene role in the process of LUAD development. It may provide a new theoretical basis for the clinical diagnosis and treatment in LUAD patients in early stage.

## 1. Introduction

Lung cancer is one of the malignant tumours with the highest incidence and mortality rate worldwide [1,2]. As the main type, lung adenocarcinoma (LUAD) accounts for nearly 40% of all lung cancer cases [3]. Patients with early-stage LUAD are usually treated by surgery but are highly susceptible to recurrence after surgery. Traditional radiotherapy and chemotherapy, on the other hand, have many side effects and are prone to radioresistance and drug resistance [4]. Despite the great advances in diagnosing and treating LUAD, the 5-year survival rate of LUAD patients remains below 20%, which is far from satisfactory [1,5,6]. The main reason for this result is the late diagnosis of LUAD, which is mostly diagnosed at an advanced stage, losing an excellent time for treatment. Therefore, finding more effective molecular biomarkers for early diagnosis and prognosis prediction of LUAD patients can help provide more objective reference indicators for treating LUAD patients and thus improve the survival rate.

The tumour immune microenvironment plays a crucial role in the treatment effect of LUAD patients. Infiltrating immune cells greatly influences the malignant progression and metastasis of tumours [7]. Neutrophils, one of the major infiltrating immune cells, are the most abundant type of immune effector cells in human blood and are the ‘first responders’ of innate immune system [8,9]. When inflammation occurs, neutrophils play a role in chemotaxis, phagocytosis, intracellular killing, extracellular capture of bacteria, and regulation of adaptive immunity [10,11]. Neutrophils highly express chemokine receptors CXCR1 and CXCR2, during the infection process, inflammatory sites release chemokines (such as CXCL1, CXCL2, CXCL5, CXCL6 and CXCL8), and neutrophils quickly enter the infected or inflammatory site through blood circulation under the recruitment of these factors [12]. In addition, inflammatory cytokines such as IL-17 and IL-1β also participate in neutrophil mobilization and recruitment [13,14]. The tumour microenvironment (TME) is also characterized by chronic inflammation. In the TME, neutrophils are classified into different types, mainly N1-type, N2-type, tumour-associated neutrophils and polymorphonuclear myeloid-derived suppressor cells [15]. Among them, N1 neutrophils exert antitumour effects and can mediate tumour killing by directly killing tumour cells or activating antitumour immunity [16,17]. In contrast, N2 neutrophils promote tumour development by rebuilding the extracellular matrix (ECM), accelerating angiogenesis and lymphangiogenesis, mediating metastasis, and immunosuppression [18,19]. Current research suggests that neutrophils may exert their pro-tumour effects mainly by forming neutrophil extracellular traps (NETs) [20,21]. NETs are meshwork structures released by activated neutrophils, mainly composed of deoxyribonucleic acid (DNA) fibres, antimicrobial proteins and histones [22]. Nuclear factor high mobility group box 1 (HMGB1) could bind to TLR4 and induce activation of the p38 MAPK/ERK signalling pathway, further leading to the release of inflammatory cytokines and resulting in the overproduction of NETs in TME [23]. In addition, activation of intravascular coagulation and microvascular thrombosis could also lead to NETs formation in TME [24,25]. NETs were found in different compartments in lung cancer. Plasma levels of NETs were found to be higher in patients with different types of tumours, including lung, pancreatic and bladder cancers, than in healthy controls [26]. The accumulation of NETs in lung tissue, peripheral blood and sputum was demonstrated in metastatic lung cancer patients [27]. On the one hand, cancer cells could induce the release of NETs from neutrophils. In mouse models of chronic granulocytic leukaemia, breast cancer and lung cancer, circulating neutrophils are more likely to release NETs than healthy animals [28]. On the other hand, NETs were related to tumour progression and metastasis. Studies have shown that NETs have both pro- and anti-cancer effects [20]. Components such as myeloperoxidase (MPO), histones and proteases in NETs can directly kill tumours and inhibit tumour growth and metastasis [29]. In recent years, more studies have confirmed that NETs promote tumour development. NETs can directly promote tumour cell proliferation through proteases or activation signals. The de-aggregated, three-dimensional DNA fibre-like network of NETs can physically trap circulating cancer cells and then promote their dissemination and metastasis [30,31]. Further, NET can dissolve the ECM via proteases (e.g. MMP9, NE), thereby promoting the release of the angiogenic factor VEGF and enhancing tumour invasion and angiogenesis [32–35]. In addition, NETs have been shown to promote tumour immune escape. NETs-DNA structure as a physical barrier has been shown to limit the contact of cancer cells with cytotoxic NK cells or T cells [36]. NETs binding proteins can also induce immunosuppression, for example, PD-L1 expression can be detected on NETs, which can lead to T-cell exhaustion [37]. Using a mouse model, Demers and Wagner found that lung cancer promoted the release of NETs and thus induced the formation of tumour thrombus, thereby worsening patients’ prognosis [38]. In addition, NETs treatment can increase mitochondrial biogenesis in tumour cells, thus promoting tumour growth [39]. However, little is known about the biomarkers related to NETs and the mechanism affecting the formation of NETs. Further analysis of NETs-related molecules is expected to provide a new strategy for prognosis prediction and treatment of LUAD patients.

Long non-coding RNAs (lncRNAs) are RNA molecules with no coding potential and transcripts longer than 200 nucleotides. lncRNAs regulate gene expression and thus influence signal transduction at multiple levels, including epigenetic, transcriptional and post-transcriptional levels [40]. In recent years, it has been found that a large number of lncRNAs are aberrantly expressed in tumours and can influence cancer development and evolution by regulating biological functions such as tumour proliferation, apoptosis and migration [41,42]. A range of lncRNAs were identified as diagnostic and prognostic markers for various malignancies, including LUAD [43,44]. The close interactions between lncRNAs and NETs have been identified. For example, lncRNA MALAT1 promotes NET formation, and NETs promote non-small cell lung cancer (NSCLC) metastasis by suppressing lncRNA MIR503HG expression to activate the inflammasome pathway [45,46]. Therefore, exploring NETs-related lncRNA signatures may help to construct new sensitive and specific prognostic predictive biomarkers for LUAD patients.

In this study, we constructed a prognostic prediction model based on public databases and datasets for LUAD patients with NETs-related lncRNAs at early stage. We also validated the sensitivity and specificity of the model for prognosis prediction in patients with early-stage LUAD patients in the follow-up analysis. In addition, we analysed the relationship of the model with tumour immunity, tumour mutation burden (TMB) and treatment sensitivity in patients with early-stage LUAD. Subsequently, this model was eventually found to serve as a new and very effective prognostic and efficacy predictive biomarker for patients with early-stage LUAD.

## 2. Materials and methods

### 2.1. Data acquisition and pretreatment

Thirty-eight NET-related genes were obtained from a literature article and summarized in the Supplementary Table 1 [47]. RNA-seq data, corresponding clinical information and simple nucleotide variation information of LUAD were downloaded from the Cancer Genome Atlas Database (https://portal.gdc.cancer.gov/), which comprised 59 normal persons and 539 LUAD patients. Patients were classified into stages I–IV according to TNM staging extracted from clinical information through R software (RStudio, Boston, MA). The stage I–II were defined as the early stage of LUAD. There were 403 patients with complete clinical information in the early stage (stage I–II) included in our final study eventually. The RNA-seq data were distinguished into lncRNA and mRNA based on gene annotation. The potential NETs-related lncRNA was screened out by co-expression analysis between NETs-related genes and lncRNA using the R ‘limma’ package at the analysis criteria of |*R*| > 0.1 and *p* < .05.

### 2.2. Construction of the prognostic NETs-related lncRNA model

The dataset, which included 403 patients, was randomly divided into training and test groups in a 1:1 ratio. In the training group, Lasso Cox regression was performed to screen the most concise lncRNA potentially based on the 1000 tenfold cross-validation. The optimal prognostic NETs-associated lncRNA were derived based on univariate and multivariate COX regressions for prognostic model construction, and a risk score was calculated for each patient using the expression values and coefficients of NETs-associated lncRNA. The following equation was calculated by:

$$\text{NETs}\;\;\text{score}=\overset{n}{\underset{i=1}{\sum }}\;\;{\text{lncRNA}}_{i}\;\;\beta_{i}$$

where *n* represents the number of lncRNAs about the risk score, *i* represents the expression value level of lncRNA *i*, and *β_i_* represents the regression coefficient of lncRNA *i* in multivariate regression analysis.

### 2.3. Evaluation and validation of NETs-lncRNA related risk score

Patients were divided into high- and low-groups according to risk scores. The prognostic value of risk scores in patients was evaluated by survival analysis curves over survival (OS) and progression-free survival (PFS). The relationship between risk score and survival status was realized through heatmap, which plotted by pheatmap package. Combining risk score with clinical characteristics of patients, univariate and multivariate Cox regression analyses were performed to determine whether risk score might be independent prognostic factors. In addition, the ROC curve was used to assess the predictive accuracy of the risk score by timeROC package. Nomogram was constructed by T-stage, N-stage, stage, age, gender and risk scores to predict the 1-year, 3-year and 5-year survival rates of patients. A calibration curve was plotted to show the consistency between prediction and actual results, and the *C*-index was used to validate the accuracy of the risk score in predicting patient survival. Subgroup analysis was used to determine whether risk scores were also significantly stratified patients in different stages.

### 2.4. Principal component analysis (PCA), functional enrichment analysis and immune-related functional analysis

PCA was used to analyse the distribution of risk scores in different populations by limma and scatterplot3d packages. Gene ontology (GO) and Kyoto Encyclopedia of Genes and Genomes (KEGG) pathway were used to explore the potential mechanism signalling pathway through clusterProfiler and enrichplot packages. The differential distribution of immune function in patients with different risk scores was analysed by gene set variation analysis package.

### 2.5. TMB analysis

The distribution of TMB in patients with different risk scores was verified and plotted by the maftool package. The correlation between TMB in two groups of patients with high and low risk scores was analysed by the limma and ggpubr packages. In addition, the relationship between survival and biomarkers (TMB and risk score) in different subgroups was analysed using the survminer package.

### 2.6. Immunotherapy analysis and estimation of drug sensitivity

Tumour immune dysfunction and exclusion (TIDE) is a computational framework to assess tumour immune escape or immunotherapy response based on pretreatment gene expression profiles of cancer samples (http://tide.dfci.harvard.edu/). TIDE data about NSCLC were downloaded from the website and use them to predict the efficacy of immunotherapy in patients with different risk scores by limma and ggpubr packages. We then screened patients with different risk scores for potential drugs and analysed drug sensitivity by the limma, ggpubr, pRRophetic and ggplot packages.

### 2.7. q-PCR analysis

RNA was extracted from the special cells and reverse transcribed into cDNA, and then the expression of target RNA was detected on the special machine according to the protocol of the SYBR Select Master Mix (Thermo Fisher, Waltham, MA), and the analysis was calculated based on the CT value 2^–ΔCt^. All primer sequences are shown in Supplementary Table 2. A549, H1975 and Beas-2B cells were obtained from the Cell Bank of the Chinese Academy of Sciences (Shanghai, China).

### 2.8. siRNA transfection

The cell transfection assay was performed as instruction of the Versatile DNA/siRNA transfection reagent (Illkirch, France, 101000046). siRNA sequences are shown in Supplementary Table 2.

### 2.9. The cell viability assay

The cell viability assay was carried out as previously described [46]. The CCK-8 kit was purchased from Beyotime (Nantong, China, C0048XL).

### 2.10. Wound-healing migration assay

Cells were seeded in six-well plates. The detection procedure was performed as previously described [46].

Images were obtained by the microscope (Olympus, Shinjuku City, Japan). The scratch damage areas at 0 h and 24 h were counted separately using ImageJ software (Bethesda, MD) to calculate the migration rate (calculation formula: wound healing rate = (0 h scratch area – 24 h scratch area)/0 h scratch area × 100%).

### 2.11. Migration assay

The migration assay was performed as previously described [46]. The crystal violet was purchased from Beyotime (Nantong, China, C0121).

### 2.12. The cell apoptosis detection

The cell apoptosis detection was performed according to the manufacturer’s instructions for the Annexin V-FITC apoptosis Detection Kit (Beyotime Biotechnology, Nantong, China, C1062L).

### 2.13. NETs isolation

The NETs isolation and handling method was performed as previously described [48].

### 2.14. Statistical analysis

All statistical analysis and image formation were performed by R software (RStudio, Boston, MA). Kaplan–Meier’s curves were used for non-parametric estimation of survival function and log-rank tests were implemented to explore statistical difference between high and low risk groups. All the experiments are the results of three or more replications. All quantitative values were obtained using the mean ± SD. *T*-test was used for comparisons between two groups, and one-way ANOVA was used for comparisons between more than two groups. All significantly statistical differences were defined as *p* < .05.

## 3. Results

### 3.1. Screening of NETs-related lncRNAs and construction of a NETs-related lncRNA prognostic model in patients with early stage LUAD

**Figure 1.**
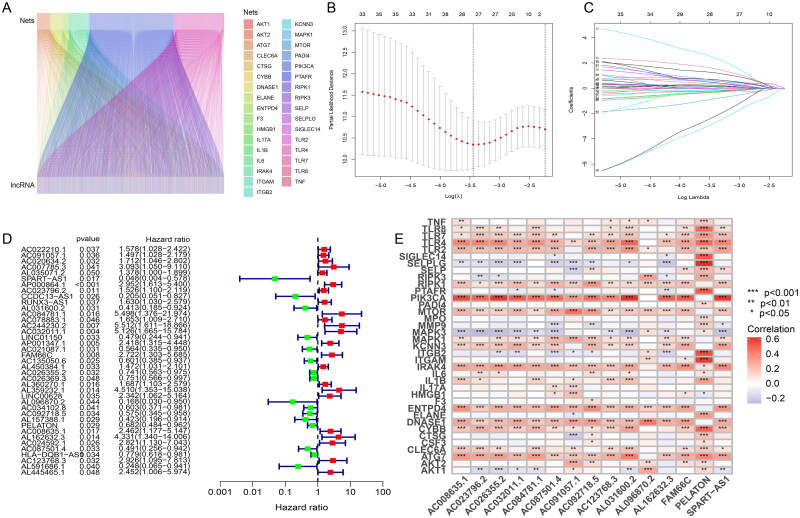
Screening of NETs-related lncRNAs in patients with early stage LUAD. (A) The Sankey plots whose lines represented co-expression relationships between genes and corresponding lncRNA; (B) Lasso Cox regression analysis for identifying the minimum number of NETs-related lncRNAs; (C) trajectory of each independent variable; (D) forest plot of NETs-related lncRNAs was screened by univariate Cox regression, as red was a symbol of high risk while green was shown as low risk; (E) the heatmap indicating the correlation between NETs-related genes and the 15 NETs-related lncRNAs, with red representing positive correlations, blue representing negative correlations, and *representing the degree of correlation between genes and lncRNAs.

When setting |*R*| > 0.1 and *p* < .05 as analysis standard, we screened 2814 NETs-associated lncRNAs through 16,876 lncRNAs and 38 NETs-associated genes (Supplementary Table 3). The co-expression association between NETs-associated genes and NETs-associated lncRNAs was shown in Sankey plots whose lines represented co-expression relationships between genes and corresponding lncRNAs (Figure 1(A)). Then, the dataset containing 403 patients was randomly divided into training and testing groups. In the training group, the minimum number of NETs-related lncRNAs was identified using Lasso Cox regression analysis and cross-validation (Figure 1(B,C)). Thirty-nine NETs-related lncRNAs were screened by univariate Cox regression, and the result was visualized in a forest plot, as red was a symbol of high risk while green was shown as low risk (Figure 1(D)). Fifteen15 NETs-related lncRNAs were identified as independent prognostic factors by multivariate Cox analysis (Supplementary Table 4). Risk scores were then calculated for each sample based on the expression values of the 15 NETs-related lncRNAs. Risk scores = (0.931984132 × AC091057.1) + (–4.121800066 × SPART-AS1) + (0.396248322 × AC023796.2) + (2.343271142 × AL031600.2) + (3.963237052 × AC084781.1) + (3.338539254 × AC032011.1) + (1.493522631 × FAM66C) + (–0.515911517 × AC026355.2) + (–5.972329416 × AL096870.2) + (–0.924106093 × AC092718.5) + (–0.723506514 × PELATON) + (1.562717474 × AC008635.1) + (1.673157591 × AL162632.3) + (–0.833083203 × AC087501.4) + (2.626810302 × AC123768.3). The heatmap indicated the correlation between NETs-related genes and the 15 NETs-related lncRNAs, with red representing positive correlations, blue representing negative correlations, and star dots representing the degree of correlation between genes and lncRNAs (Figure 1(E)). In summary, 15 NETs-related lncRNAs were screened lastly and the risk scores were quantified and calculated based on the expression of the 15 NETs-related lncRNAs for the follow-up study.

### 3.2. Validating the prognostic predictive value of this NETs-associated lncRNA signatures

**Figure 2.**
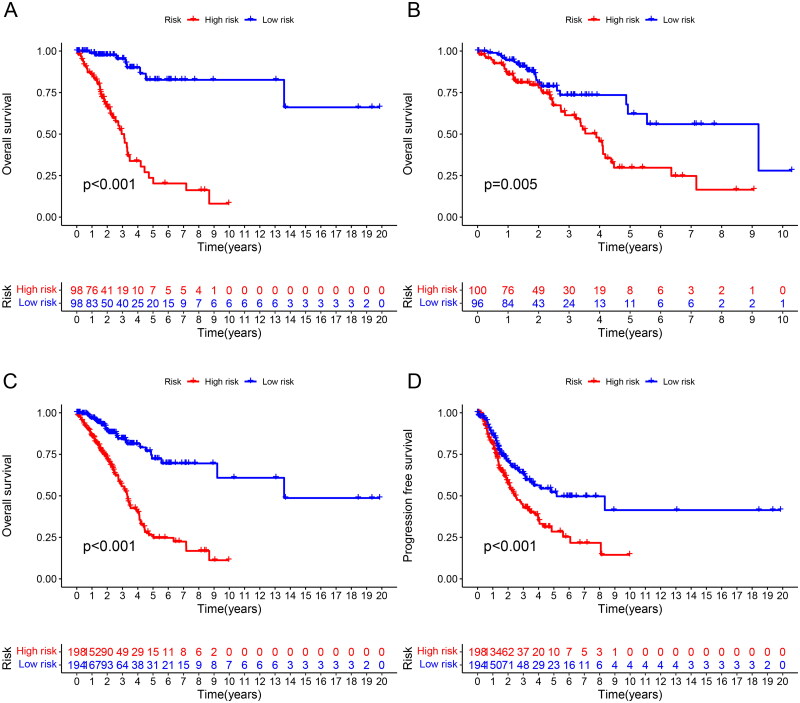
Survival analysis of the prognostic value of NETs-related lncRNA signature. (A) OS curve about the high- and low-risk scores in the training group; (B) OS curve about the high- and low-risk scores in the testing group; (C) OS curve about the high- and low-risk scores in all groups; (D) PFS curve about the high- and low-risk scores in all groups.

**Figure 3.**
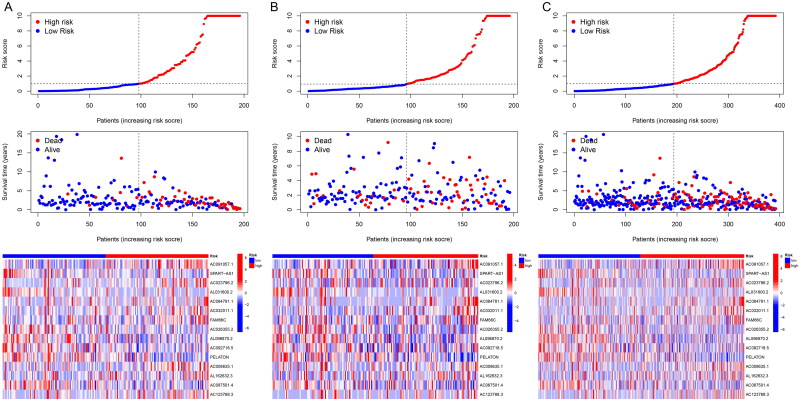
Prognostic risk model evaluation. (A–C) Risk curve reflects the association between risk scores and survival status in patients with early stage LUAD. The heatmap indicated characteristic distributions of the lncRNAs. (A) Training group; (B) testing group; (C) all groups.

To better evaluate the prognostic value of risk scores signature, patients included were divided into high-risk and low-risk groups on the basis of median value. The OS and PFS were significantly longer in the low-risk group than in the high-risk group among the training, test and all sets (Figure 2(A–D)). The association between risk scores and survival status in LUAD patients with early stage was reflected in the risk curves, and it was demonstrated that higher mortality occurred in high-risk patients than low-risk patients. Heatmap showed the expression of 15 NETs-related lncRNAs in high and low risk groups (Figure 3(A–C)). So, we could conclude that the risk score signature was significant in the prediction of patient prognosis.

### 3.3. Construct a nomogram to verify the independent prognostic value of the risk scores signature

**Figure 4.**
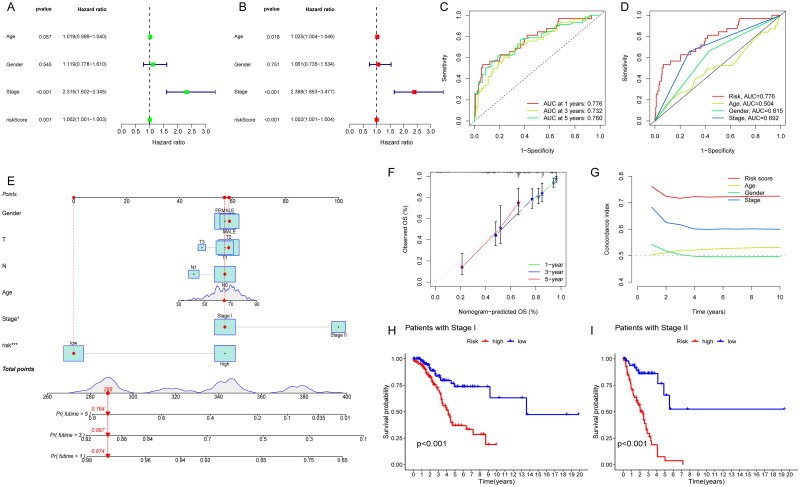
Construction of a nomogram combined risk score and clinical indicators for predicting survival of LUAD patients. (A) Univariate Cox regression analyses based on the risk score and corresponding clinical characteristics; (B) multivariate Cox regression analyses based on the risk score and corresponding clinical characteristics; (C) time ROC curves for 1-year, 3-year and 5-year OS; (D) clinical characteristic ROC curves for comparing risk score and other factors; (E) nomogram plot model construction for predicting the OS in patients with early stage LUAD; (F) the 1-year, 3-year and 5-year calibration curves; (G) the *C*-index analysis; (H) subgroup OS analysis in patients with stage I; (I) subgroup OS analysis in patients with stage II.

To explore whether this signature could predict the survival of patients independently, which might be prior to other clinical characteristics, we performed univariate and multivariate Cox regression analyses. The results revealed that stage (HR = 2.398, 1.653–3.477, *p < .001*) and risk score (HR = 1.002, 1.001–1.004, *p < .001*) was independently relevant to OS, indicating that risk score is an independent prognostic factor in patients with early stage LUAD (Figure 4(A,B)). In addition, the ROC curves were plotted to evaluate the predictive accuracy of the risk score. The AUCs for 1-year, 3-year and 5-year OS were 0.776, 0.732, and 0.760, respectively (Figure 4(C)). The AUC for risk score (0.776) was better than those for age (0.504), gender (0.615) and stage factors (0.692) (Figure 4(D)). Then, we established a nomogram combining clinical characteristics (age, gender, TNM stage, T stage, N stage) with risk score for the purpose of quantitative detection about OS and risk score. It provided a readable measurement in predicting 1-, 3- and 5-year OS in patients with early stage LUAD (Figure 4(E)). The 1-year, 3-year and 5-year calibration curves matched the standard one nearly, demonstrating that the predicted survival of this nomogram was in close agreement with the actual survival (Figure 4(F)). Additionally, it included that higher *C*-index values for the risk score than for other clinical characteristics, such as age, gender and stage (Figure 4(G)). The results above indicate that risk score had high predictive accuracy and reliability for clinical diagnosis. Subgroup survival analysis was calculated to observe the predictive significance in patients with high- or low-risk groups at stage I and II (*p < .05*). It showed that low-risk patients had longer OS than high-risk group, which suggested that the risk score could stratify and screen patients significantly in clinical (Figure 4(H,I)). In brief, the nomogram combining clinical characteristics (age, gender, TNM stage, T stage, N stage) with risk score had an advantage in the detection of OS, and the risk scores may be helpful in recognizing patients with worse prognosis.

### 3.4. PCA, functional enrichment analysis and immune-related functional analysis of the risk score signature

**Figure 5.**
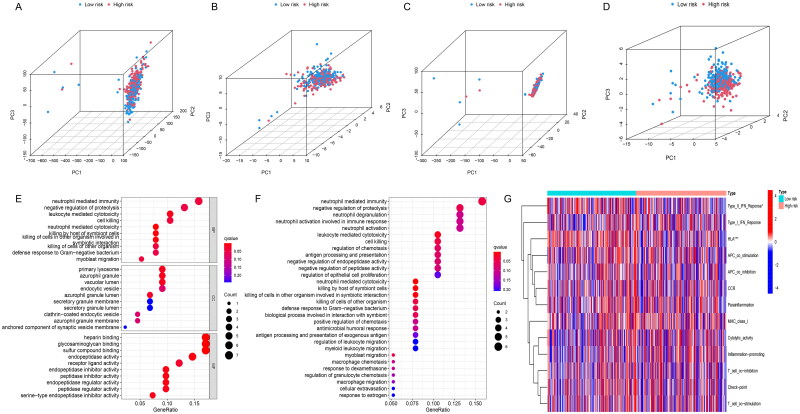
The exploration of the distribution and the potential functional pathways of risk score-related lncRNAs. Principal component analysis in (A) all genes; (B) NETs-related genes; (C) all lncRNAs related with NETs; and (D) risk score-related lncRNAs; (E) GO enrichment analysis about the risk score; (F) KEGG enrichment analysis about the risk score; (G) immune-related functional analysis about the risk score.

PCA analysis was to explore the spatial dimensional distribution of all genes, NETs-related genes, NETs-related lncRNAs and risk score-related lncRNAs, and the results showed a better and clearer hierarchical clustering of risk score-related lncRNA. It shows that risk score is more reliable in model establishment and makes a good performance in distinguishing between two groups (Figure 5(A–D)). In addition, we used GO and KEGG enrichment to investigate the potential mechanisms of risk score model associations. GO analysis indicated risk score-related lncRNAs were closely associated with the neutrophil-mediated immunity, negative regulation of proteolysis, leukocyte-mediated cytotoxicity, cell killing (Figure 5(E)). KEGG analysis resulted that 15 NETs-related lncRNAs signatures were enriched in partial pathways in GO analysis and neutrophil degranulation, neutrophil activation involved in immune response, neutrophil activation, leukocyte mediated cytotoxicity (Figure 5(F)).

Neutrophil activation and neutrophil-derived chemokines were related to the immune system [49]. We analysed the distribution of immune-related functional status in high- and low-risk patients, and it showed that the expression of type II interferon (IFN) response and human leukocyte antigen (HLA) was significantly lower in patients in the high-risk group compared to those in the low-risk group, while other immune functions had no statistical significance (Figure 5(G)). A previous study reported that NETs could stimulate the release of cytokines from dendritic cells and macrophages, leading to T cell activation and expansion of immune cell recruitment [50]. Whether IFN and HLA have an effect on the function and expression of NETs and lncRNAs via immune cells is unknown. The relationship between 15 NETs-related lncRNAs and tumour immune microenvironment need future exploration. In summary, risk-score signature can better distribute patients spatially and has effects on tumour progression and tumour immune microenvironment, but further experimental validation is still needed.

### 3.5. The correlation between risk score and TMB or drug sensitivity

**Figure 6.**
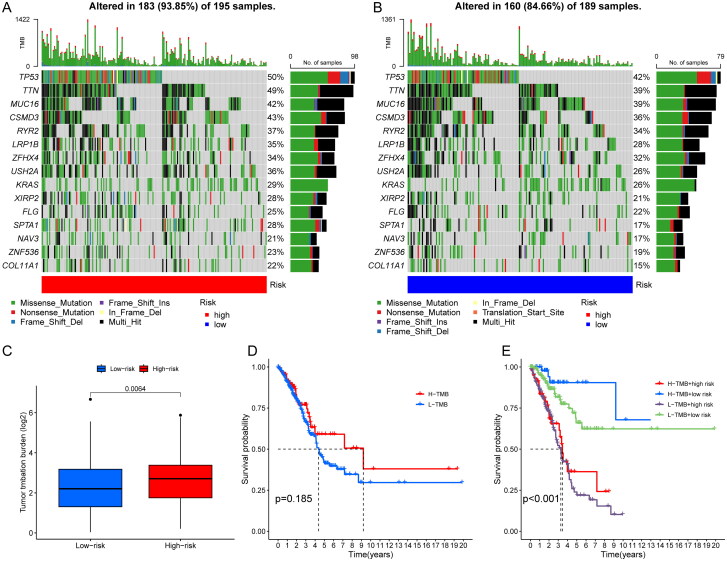
Correlation analysis of TMB and risk score. (A, B) Waterfall plot identifying comparing the frequency of mutations of the top 15 highest mutated genes in the high-risk group (A) and in the low-risk group (B). (C) comparison about the TMB in high- and low-risk groups. (D) OS analysis in different TMB groups; (E) OS analysis in different TMB and risk scores groups.

**Figure 7.**
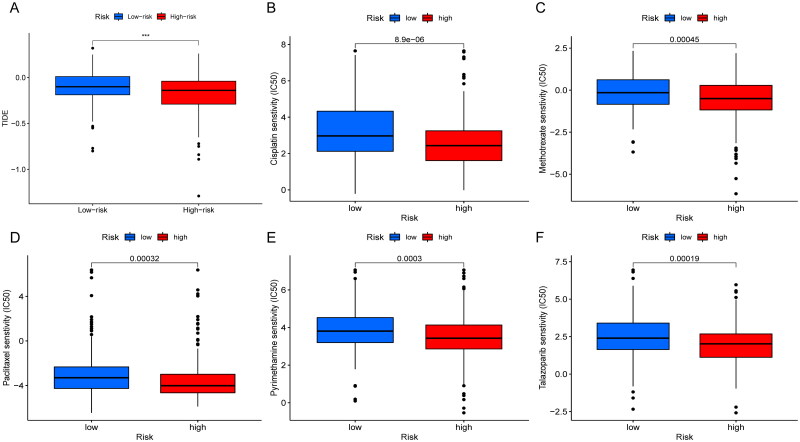
Correlation analysis of immunotherapy and drug sensitivity with risk score. (A) TIDE analysis in different risk scores groups; (B–F) drug sensitivity analysis of cisplatin (B), methotrexate (C), paclitaxel (D), pyrimethamine (E), talazoparib (F) in high- and low-risk group patients.

The mutations were calculated separately for both high and low risk groups by the maftools algorithm. Comparing the top 15 highest mutated genes, the high-risk group was associated with more elevated frequency of mutations than those in the low-risk group as shown in the graph (TP53: 50% vs. 42%. TTN: 49% vs. 39%; MUC16: 42% vs. 39%) (Figure 6(A,B)). And the TMB relationship in high- and low-risk groups was statistically significant (*p = .0064*) (Figure 6(C)). When patients were divided into two groups of high and low TMB, there was no statistical difference in OS. Then, we combined the two biomarkers of risk score and TMB and divided the patients into four groups: high TMB and high risk, high TMB and low risk, low TMB and high risk, and low TMB and low risk. The results suggested that OS was significantly longer in patients with high TMB and low risk. When we observed the TMB of patients in the risk group, the OS was significantly higher in the high TMB group than in the low TMB group (*p < .05*) (Figure 6(D,E)). We then explored the differences in response to immunotherapy among patients in different risk groups. The higher the TIDE, the better the efficacy of immunotherapy. We found that the TIDE was higher in the low-risk group than in the high-risk group, indicating that patients in the low-risk group were more sensitive to immunotherapy, but further exploration and validation are needed to investigate whether patients in the low-risk group actually have better immunotherapy efficacy in clinical (Figure 7(A)). The pRRophetic package was used to screen potential sensitive anti-tumour drugs containing cisplatin, paclitaxel, methotrexate, pyrimethamine, talazoparib, etc. The results showed that lower IC_50_ was more common in the high-risk group, suggesting that patients in the high-risk group might be more sensitive to these drugs (Figure 7(B–F)). In a word, the risk scores showed significant patient screening ability on patients with high TMB. Patients with high-risk scores may benefit from cisplatin, paclitaxel, methotrexate, pyrimethamine and talazoparib, which may assist in patient medicine guidance.

### 3.6. NETs promote the metastasis of NSCLC

**Figure 8.**
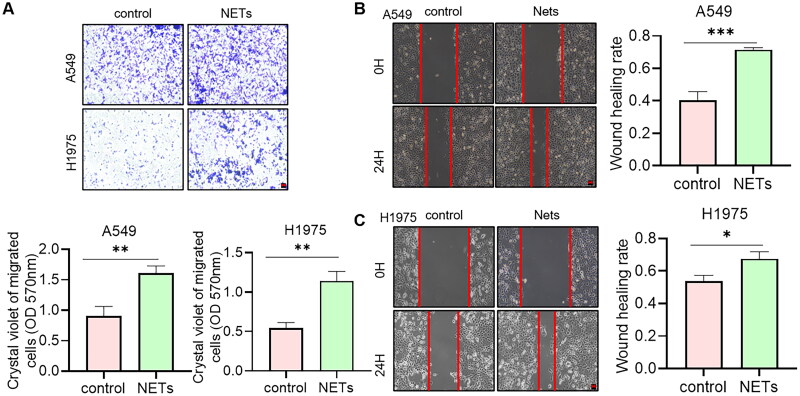
(A) Transwell assays for the effect of NETs on the migration ability of A549 and H1975 cells. Scare bar: 200 μm, *n* = 3. The absorbance value at 570 nm was measured after washing the cells through Transwell membrane with 33% glacial acetic acid to quantify the migration ability of the cells. (B, C) Scratch assay to detect the effect of NETs on the migration ability of A549 and H1975 cells. Scare bar: 200 μm, *n* = 3. *, ** and ***, represent *p* < .05, *p* < .01 and *p* < .001, respectively. All quantitative values were obtained using the mean ± SD.

The data above indicated that NETs affected the prognosis of NSCLC patients. So, whether NETs can affect tumour progression at the cellular level was explored. NETs were acquired from blood to stimulate tumour cells. Crystalline violet staining showed that the number of cells crossing the Transwell membrane was significantly higher under the condition of NETs (Figure 8(A)). Meanwhile, the scratch healing ability was significantly increased in NETs group (Figure 8(B,C)). We could conclude that NETs influenced the migration ability of NSCLC in cell level. Blocking NETs-related pathways may have potential impact in the treatment of tumours.

### 3.7. FAM66C promotes NSCLC development

**Figure 9.**
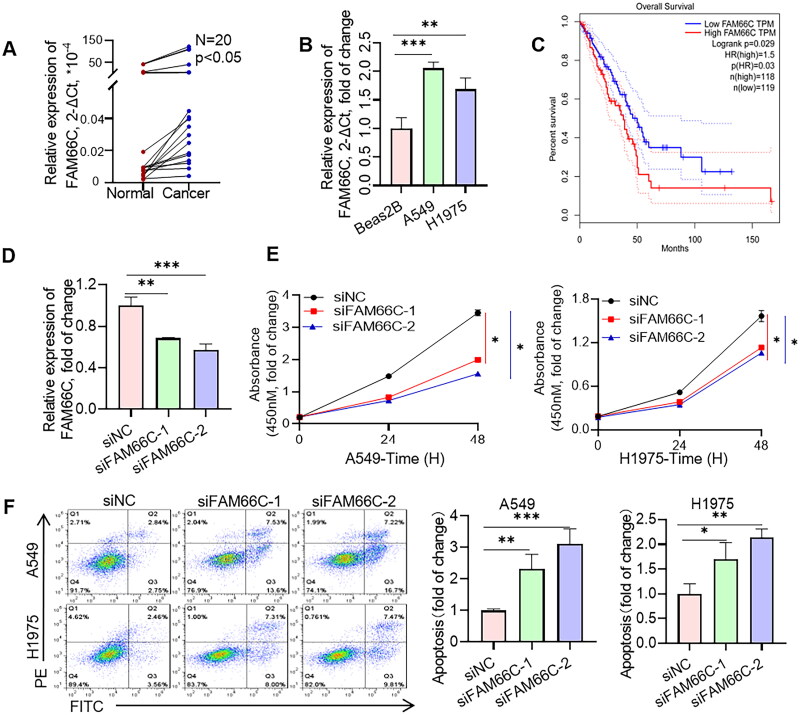
Experiments validate the role of FAM66C in lung cancer. (A) Detection of FAM66C mRNA level in NSCLC tumour and adjacent normal tissues by qPCR, *n* = 20; (B) detection of FAM66C mRNA level in NSCLC cell lines and normal lung bronchial epithelial cell Beas-2B, *n* = 3. (C) Kaplan–Meier’s survival curve about early-stage LUAD patients using TCGA data. (D) Detection of siRNA knockdown efficiency in A549 cell line, *n* = 3. (E) CCK8 detection of the effect of FAM66C knockdown on the proliferative capacity of A549 and H1975 cell lines, *n* = 3. (F) Flow cytometry detection of apoptosis in A549 and H1975 cells treated with FAM66C knockdown, *n* = 3. *, ** and ***, represent *p* < .05, *p* < .01 and *p* < .001, respectively. All quantitative values were obtained using the mean ± SD.

**Figure 10.**
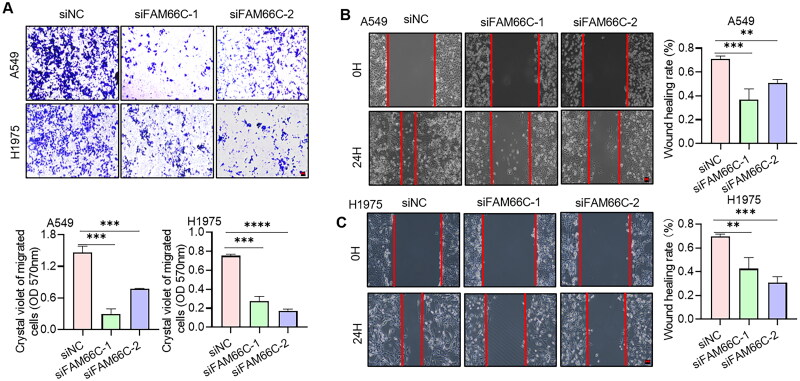
(A) Transwell assays for the effect of FAM66C knockdown on the migration ability of A549 and H1975 cells. Scare bar: 200 μm, *n* = 3. The absorbance value at 570 nm was measured after washing the cells through Transwell membrane with 33% glacial acetic acid to quantify the migration ability of the cells. (B, C) Scratch assay to detect the effect of FAM66C knockdown on the migration ability of A549 and H1975 cells. Scare bar: 200 μm, *n* = 3. All quantitative values were obtained using the mean ± SD.

To further investigate the role of these lncRNAs in NSCLC progression, we chose the well-studied FAM66C for further functional studies. We first analysed the transcript levels of FAM66C in tumour and paraneoplastic tissues of 20 NSCLC patients by qPCR assay. The results indicated that the mRNA level of FAM66C was significantly increased in the cancer tissues of NSCLC patients (Figure 9(A)). We also found that the expression of FAM66C was upregulated in lung cancer cell lines compared to normal lung bronchial epithelial cells (Figure 9(B)). In addition, Kaplan–Meier’s analysis showed that lower FAM66C were associated with longer OS through TCGA database (Figure 9(C)). From the above, we found that FAM66C was an oncogene and has a significant correlation with the prognosis of lung cancer, and we found that FAM66C could promote the proliferation and migration in tumour cells through screening previous literature [51]. However, the role of FAM66C in lung cancer remains unknown. In order to validate the hypothesis above, the siFAM66C sequences were obtained from the previous literature [52], we performed siRNA transfection on A549 cell line, qPCR was performed to detect siRNA knockdown efficiency. The results showed that both siRNA sequences had an interfering effect of FAM66C expression (Figure 9(D)). Therefore, we selected these two sequences for subsequent experiments. When FAM66C was knocked down in A549 and H1975 cells, the CCK8 assay showed that the proliferation ability was reduced (Figure 9(E)). Thus, we tried to explore whether the FAM66C affects the proliferation of tumour cells, the results of apoptosis assay showed an increased percentage of apoptosis of cells after knockdown of FAM66C, including early and late apoptosis (Figure 9(F)). Additionally, the FAM66C interference effect on migratory ability in lung cancer cells was verified by wound-healing assay and migration assay. Crystalline violet staining showed that the number of cells crossing the Transwell membrane was significantly lower in the FAM66C knockdown group than in the control group (Figure 10(A)). Moreover, the scratch healing ability was significantly inhibited in FAM66C knockdown of A549 and H1975 cells (Figure 10(B,C)). The results above suggested that FAM66C may play an oncogene role in the process of NSCLC development, and FAM66C knockdown significantly influenced the prefiltration and migration ability of the tumour cells.

## 4. Discussion

As the most common type of lung cancer, patients with LUAD have a very poor prognosis [53]. The current risk signature constructed based on tumour essence does not work well. Therefore, it is important to construct more reliable predictive models to accurately assess the prognosis and survival of patients with LUAD [54]. NETs are DNA-based reticulation structures produced by neutrophils after activation and inlaid with various granular proteins, neutrophil elastase and other contents [55]. NETs have been shown to awaken dormant tumour cells, which in turn promote tumour recurrence and metastasis [56]. There is also evidence that NETs influence tumour progression by modulating the immune system [29]. NETs are considered as a promising target for cancer treatment. On the other hand, as novel biomarkers, lncRNAs can play both pro- and oncogenic roles in tumours [41]. Abnormal expression and function of several lncRNAs have been reported to be closely associated with the progression of LUAD [57]. For the relationship between lncRNAs and NETs, it has been shown that lncRNAs were bidirectionally regulated with NETs [45,46]. On this basis, we constructed a prognostic model based on NETs-related lncRNAs to predict the prognosis and tumour-related characteristics of LUAD patients.

In this study, NETs-related lncRNAs were obtained by building a co-expression network between lncRNAs and NETs-related genes. Then, using univariate and multivariate Cox regression analyses, we obtained a total of 15 prognostic NETs-related lncRNAs. With these 15 lncRNAs, we constructed a prognostic signature and divided the patients into high- and low-risk groups based on the median value of the risk scores. Survival analysis showed that the prognostic characteristics of LUAD patients in the high-risk and low-risk groups could be accurately distinguished. Patients in the high-risk group had higher mortality and shorter survival. Moreover, univariate and multivariate Cox regression analyses confirmed that this NETs-related lncRNAs signature was prognostic factors independent of other common clinical characteristics. Using stratified analyses, we found that the NETs-related lncRNAs signature can clearly distinguish between high- and low-risk patients at different stages. These findings suggested that the 15-lncRNA signature is highly sensitive and specific biomarker for predicting the clinical outcomes of patients with LUAD.

Among these 15 NETs-related lncRNAs, FAM66C has been shown to interact with and inhibit the expression of miR-574-3p, inhibiting pancreatic cell proliferation and promoting their apoptosis [58]. FAM66C acts as a tumour suppressor in glioma by targeting miRNA/LATS1 signalling [52]. In contrast, FAM66C is elevated in prostate cancer, and high FAM66C expression promotes tumour growth by activating EGFR-ERK signalling through inhibition of the proteasome pathway [59]. FAM66C was also significantly increased in intrahepatic cholangiocarcinoma. FAM66C drives intrahepatic cholangiocarcinoma progression and glycolysis by sponging miR-23b-3p and thus upregulating KCND2 expression [60]. In addition, FAM66C has been incorporated into prognostic models for melanoma [61], gastric cancer [62] and glioblastoma (GBM) [51]. However, functions of FAM66C in lung cancer remain elucidated. Here, we found for the first time that FAM66C is upregulated in NSCLC and associated with poor prognosis. The results of cellular experiment validation revealed that FAM66C was closely associated with tumour proliferation and migration. Yet, the mechanism it regulates its oncogenic role in lung cancer needs further investigation. PELATON, also known as LINC01272, has been shown to regulate the epithelial mesenchymal transition process in colorectal and gastric cancers [63,64]. In GBM, PELATON inhibited p53 expression in p53 wild-type GBM cells, and knockdown of PELATON inhibited the proliferation and migration of GBM cells [65]. It is reported that PELATON is a biomarker of lung cancer [66–68]. Low expression of PELATON is related to the poor prognosis of patients [69]. Nevertheless, the potential pathways or molecular targets influencing lung cancer are unknown. In addition, lncRNA AC091057.1 and AC026355.2 have been included in various LUAD prognostic models, but their specific roles are unknown [70–74]. SPART-AS1 is a novel RNA whose prognostic value in tumours has not yet been investigated. However, further studies are needed regarding the potential role or mechanism of SPART-AS1 in regulating the malignant progression of NSCLC. The roles and prognostic value of the rest lncRNAs in tumours have not been previously investigated. Uncovering the functions of these newly identified NETs-related lncRNAs may help to understand the pathogenesis and progression mechanisms of LUAD, thus providing new targets for treating LUAD patients. KEGG analysis revealed that the significant difference in prognosis between the high-risk and low-risk groups may be related to neutrophil-mediated immune responses and leukocyte-mediated cytotoxicity, a result that is predictable. These functional enrichment analyses also provide a direction for exploring the functions of these lncRNAs in the future. In addition, although we clarified that these 15 genes are lncRNAs associated with NETs, there are no relevant studies on how they affect or are affected by NETs. Therefore, it is necessary to carry out more research to clarify how these lncRNAs link with NETs to affect the prognosis of LUAD patients.

We also analysed the relationship between immune-related functions, TMB and risk scores in early-stage LUAD patients. It concluded that type II-IFN response and HLA were inhibited in low-risk patients. Type II-IFNs are central coordinators of the immune response and have both anti-tumour and pro-tumour capabilities [75]. HLA is an independent factor in tumour-associated antigen presentation and is involved in the process of tumour immune escape [76]. These differences in immune characteristics suggest that our signature may be involved in regulating the tumour immune microenvironment in LUAD, which may contribute to the poorer prognosis of high-risk patients. Then, we analysed the high- and low-risk group’s mutation, and found that missense mutation was the most common mutation type in both groups. The mutation frequencies of the top 15 most commonly mutated genes in LUAD were all higher in the high-risk group of patients, including the best-known tumour suppressor TP53. Tumour cells with a high frequency of gene mutations carry more tumour antigens on the cell surface and are more likely to be recognized and attacked by the immune system; therefore, TMB has been proposed as a biomarker for predicting response to immunotherapy [77,78]. We discovered that the TMB value of high-risk group was higher than that of low-risk group, suggesting that high-risk patients may be more sensitive to immunotherapy. Notably, TMB alone had no predictive value for the prognosis of early stage LUAD patients, but superimposing our signature with TMB showed good prognostic predictive value. TIDE is a computational framework that can effectively predict the efficacy of immune checkpoint inhibitors (ICIs) therapies and, as previously mentioned, the higher the TIDE score, the greater the possibility of tumour immune escape, and the less likely the patient will benefit from ICIs [79]. Our analysis showed that patients in the high-risk group had lower TIDE scores, suggesting that high-risk patients may be more sensitive to ICIs treatment, a parsing result consistent with TMB. Drug sensitivity analysis revealed that high-risk group patients were more sensitive to a range of targeted and chemotherapeutic agents. All the above analyses can guide the use of drug therapy in patients with early-stage LUAD.

Although the signature has good prognostic value, this study still has some limitations. First, tumour heterogeneity makes sampling unavoidably biased and external validation of other independent LUAD cohorts is necessary. Second, the signature was constructed as a retrospective study based on database analysis, and it was essential to verify its validity and usefulness with prospective studies. In addition, the lack of corresponding functional and mechanistic studies has resulted in the impossibility of understanding the role played by NETs-related lncRNAs in LUAD progression.

## 5. Conclusions

In conclusion, in this study, we constructed a NETs-related lncRNAs signature that shows great potential to predict prognosis, TMB, immunotherapy and drug sensitivity of LUAD patients. FAM66C plays an oncogene role in NSCLC development by promoting lung cancer cells’ proliferation and migration capacity. This work provides a new theoretical basis for diagnosing and treating LUAD patients.

## Supplementary Material

Supplemental Material

## Data Availability

All datasets generated for this study are included in the article/supplementary material.
